# Spatial Attention Fusion for Obstacle Detection Using MmWave Radar and Vision Sensor

**DOI:** 10.3390/s20040956

**Published:** 2020-02-11

**Authors:** Shuo Chang, Yifan Zhang, Fan Zhang, Xiaotong Zhao, Sai Huang, Zhiyong Feng, Zhiqing Wei

**Affiliations:** 1 School of Information and Communication Engineering, Beijing University of Posts and Telecommunications, Beijing 100876, China; zhangyf@bupt.edu.cn (Y.Z.); zhaoxiaotong@bupt.edu.cn (X.Z.); huangsai@bupt.edu.cn (S.H.); fengzy@bupt.edu.cn (Z.F.); weizhiqing@bupt.edu.cn (Z.W.); 2 School of Information and Communication Engineering, Beijing Information Science and Technology University, Beijing 100101, China.; zhangfan2015@bupt.edu.cn

**Keywords:** autonomous driving, obstacle detection, mmWave radar, vision, spatial attention fusion

## Abstract

For autonomous driving, it is important to detect obstacles in all scales accurately for safety consideration. In this paper, we propose a new spatial attention fusion (SAF) method for obstacle detection using mmWave radar and vision sensor, where the sparsity of radar points are considered in the proposed SAF. The proposed fusion method can be embedded in the feature-extraction stage, which leverages the features of mmWave radar and vision sensor effectively. Based on the SAF, an attention weight matrix is generated to fuse the vision features, which is different from the concatenation fusion and element-wise add fusion. Moreover, the proposed SAF can be trained by an end-to-end manner incorporated with the recent deep learning object detection framework. In addition, we build a generation model, which converts radar points to radar images for neural network training. Numerical results suggest that the newly developed fusion method achieves superior performance in public benchmarking. In addition, the source code will be released in the GitHub.

## 1. Introduction

**Figure 1:**
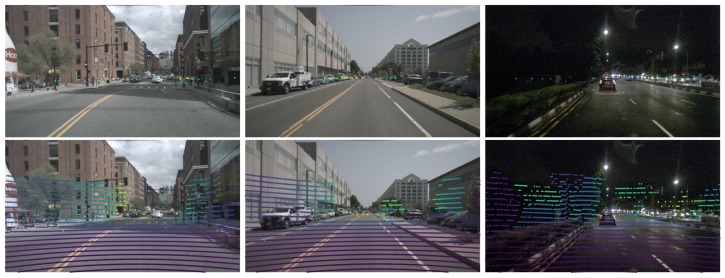
The rendering results in the camera coordinate about radar points (top row) and LiDAR points (bottom row). As shown in the figure, the return number of radar points is sparse compared with the LiDAR points. In addition, points in the camera coordinate with different colors represent different depth values.

In autonomous system applications, an accurate understanding of the surrounding environment under all conditions is important for safety consideration. In general, the autonomous vehicles need to accurately estimate the positions of obstacles, and make decisions for path planning. The input data of autonomous vehicles is from various sensors, such as mmWave radar, vision sensor, and LiDAR, which are used by carefully designed recognition algorithms to make perception. However, the properties and prices of different sensors are various. In practice, we should consider their differences to design an applicable fusion scheme. Some of rendering examples in the image planes about radar points and LiDAR points are depicted in Figure 1.

For the vision sensors, the appearance information of targets can be preserved. They can also provide accurate lateral measurements. For safety consideration, the perceptual algorithm should infer the 2D position of obstacle from vision image in all scales. However, small targets occupy very few pixels in an image, which are susceptible to background noise [1,2,3,4,5,6]. Although the feature pyramid-based detection model can improve the detection accuracy in small objects [7,8,9,10], it is far from perfect for safety and practical consideration in autonomous driving. Moreover, the appearance of obstacles is easily blurred by the bad weather such as rain, snow, fog, night, etc., which is difficult for the perceptual algorithm to distinguish the target from background.

In contrast to vision sensors, the millimeter wave radar (mmWave radar) using short-wavelength electromagnetic waves is a special class of radar technology, which is common in autonomous driving. This radar system emits an electromagnetic wave signal that is then reflected by object in its propagation path. By capturing the reflected signal, the radar system can determine range, velocity, and angle of object. The mmWave radar has a longer detection range compared to LiDAR. Moreover, the mmWave radar penetrates fog, smoke, and other obscurants much better than infrared sensors. By the way, mmWave radar is cheaper than LiDAR. However, the mmWave radar has too few radar points to effectively highlight the boundaries of obstacles. Furthermore, the mmWave radar has limited lateral resolution. When exploiting the advantages of vision sensor and mmWave radar, these two kinds of sensors complement each other.

**Figure 2:**
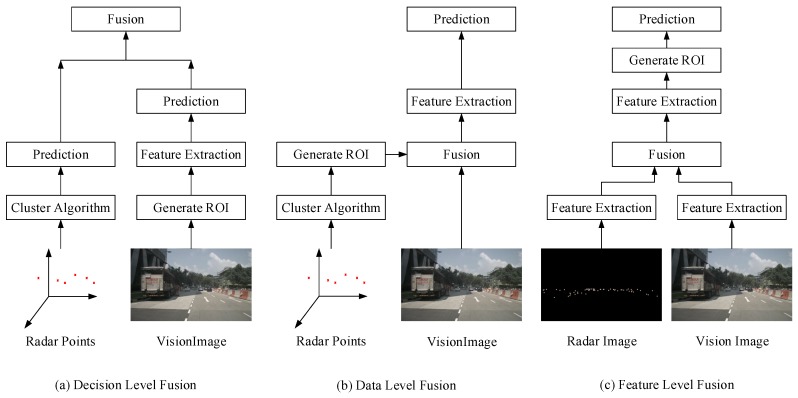
Three different fusion schemes using mmWave radar and vision sensor for obstacle detection.

Hence, it is a promising direction for obstacle detection with the fusion of mmWave radar and vision sensor. In general, there are mainly three different fusion schemes using mmWave radar and vision sensor, which are shown in Figure 2. They are decision level fusion, data level fusion and feature-level fusion, respectively. Moreover, the speckles on the radar images shown in Figure 2 are the detection signals returned by the radar sensor itself. These radar images are produced by our redesigned radar image generation model inspired by the work of [11].

The first fusion scheme works in decision level. The prediction results from radar and vision sensor are fused to generate the final results [12,13,14,15,16,17,18,19,20,21,22,23,24]. However, there are different kinds of detection noises involved in these two kinds of prediction results. Thus, it is difficult to model a joint detection probability density function, which is important to design a fusion filter algorithm in the decision level. In practice, the detection performance improvement with decision level fusion is limited, and it is computationally expensive.

For the data level fusion scheme [25,26,27,28,29,30,31,32,33,34], first, we can generate the region of interests (ROIs) based on radar points in the camera coordinate. Then, we extract the corresponding image patches in the input vision image based on the generated ROIs. Finally, we can use the feature-extraction model and classification model to determine whether there is an obstacle in the extracted image patch. There is no doubt that the detection recall rate is impacted by the number of effective radar points. When a subregion of image plane has no radar point, the obstacles in that area cannot be detected because they are completely ignored by the whole detection pipeline depicted in data level fusion scheme. Thus, the theoretical value of the object detection recall rate is limited. Although the data level fusion scheme can reduce the search region in the camera coordinate according to the information of mmWave radar, which save computing resources. Therefore, it is not a good idea to take the data level fusion scheme in the autonomous system for safety consideration.

As for the feature-level fusion scheme [11,35,36], it is a recent popular fusion methodology. In general, the feature-level fusion scheme transforms radar points from the 3D world to a 2D image plane. The depths and velocities reflected by radar points are stored as pixel values in the transformed radar image. The generated radar image examples are depicted in Figure 2. It has multi-channels, and different channels correspond to different physical states of surroundings, which are measured by the radar sensor. Thus, we can get two kinds of images for the same driving scene: radar image and vision image. More importantly, based on the vision image and radar image, the CNN can be used to extract information from both. The designed CNNs detection models using the feature-level fusion scheme can learn the relationship between radar and vision data, which are the most effective method to use both of the radar and vision information.

In the pioneering work of Simon et al. [11], the CNN detection model using the feature-level fusion scheme was first presented, where the framework of the detection model was inspired by the SSD [2] detection method. The feature-level fusion scheme [11] was the element-wise add operation between radar feature map and vision feature map. However, the element-wise add operation was not suitable for heterogeneous feature maps, which was confirmed in our experiments. Moreover, to deal with the sparsity of radar points, the authors designed an automatic generation model to transform the radar information to a 3D matrix, which was easily used as the input data of CNN detection methods. However, the details about the radar image generation model [11] were not clear. In this paper, we reproduce that generation model and make some changes about how to convert the physical states in radar points to corresponding pixel values in the radar image. In addition, we do a lot of experiments to select the optimal hyperparameters in the radar image generation model, which is based on the improvement of detection performance. Inspired by the CNN-based feature fusion scheme, John et al. [35] also proposed a CNN feature fusion detection model based on the YOLO [1] detection framework. More importantly, in the work of [35], the authors proved that the feature fusion scheme using radar and vision outperformed the other fusion schemes. In the work of [36], the authors proposed a CameraRadarFusionNet (CRF-Net) to learn at which level the fusion of the sensor data was the most beneficial for the detection task. Furthermore, they introduced a training method called BlackIn to ensure the convergence in model training.

In contrast to the above schemes [11,35,36], in this paper, we propose a spatial attention fusion (SAF) method in the feature level, which can effectively merge the radar feature maps and vision feature maps. As we all know, radar points reflect the physical states of an autonomous vehicle’s surrounding environment. Thus, if the vision image areas are corresponding to radar points, obstacles occur with high probability. Based on that, we design a CNN sub-network to extract spatial attention information as control signals to fuse the vision feature maps. The input data of the sub-network is radar images, which are produced by our improved radar image generation model. For the SAF, it is mainly made up of convolution layers with different receptive fields. To determine the most effective mix of convolution layers, we conduct lots of experiments using different SAF configurations on the validation set. For the detection speed consideration, we build the SAF upon the fully convolutional one-stage object detection framework (FCOS) [10], which is very popular in recent detection community for its simple idea and high accuracy.

**Figure 3:**
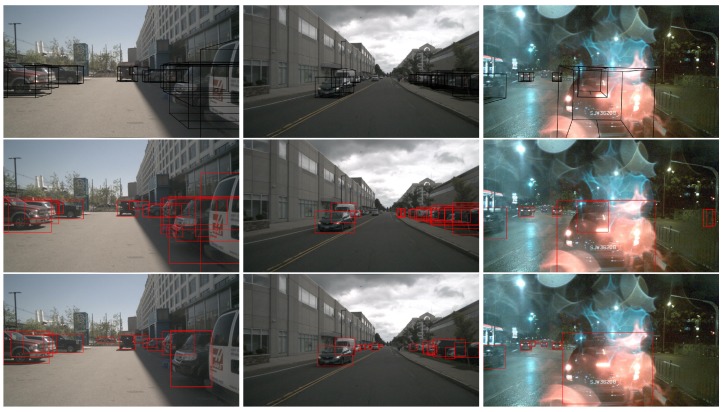
The annotations of front camera in nuScenes dataset [37]. **Top row**: the original annotations provided by the nuScenes, which are 3D bounding boxes colored by black. **Middle row**: the generated 2D annotations by converting the 3D bounding boxes. **Bottom row**: the 2D annotations generated by our proposed method.

For the training data, the dataset of nuTonomy scenes (nuScenes) [37] is used. As suggested by the nuScenes [37], the nuScenes dataste is split as training dataset, validation dataset and test dataset, which have 700 scenes, 150 scenes and 150 scenes, respectively. However, the labeled bounding boxes are 3D, and they are not proper for the 2D detection framework FCOS, which is used as detection backbone in this paper. As a result, we should convert the 3D labeled bounding boxes to 2D annotations. Some of results are depicted in Figure 3.

As shown in the top row of Figure 3, we can notice that the vehicles are still labeled even though they are obscured by other vehicles. After converting the 3D bounding boxes to 2D annotations, there are more than one bounding boxes in a vehicle, which are confused annotations for CNN model learning. To deal with that, we generate the 2D annotations by enhanced version of FCOS with the ResNet-101 as feature-extraction backbone (The training configure file of FCOS model for generating 2D annotations is fcos_imprv_dcnv2_X_101_64x4d_FPN_2x.yaml, which has 46.6 AP in minival of COCO dataset [38]. For more details about FCOS model [10], please visit the GitHub [39]), which has very slow running speed and very high accuracy. Moreover, all generated 2D annotations by FCOS are roughly examined and adjusted by human. Similar to the work of [11], we only keep the generated 2D annotations with class labels of *bicycle, car, motorcycle, bus, train, truck*, which are collectively identified as *obstacle* category. As a result, there are six classes of objects are treated as obstacles in road. As for pedestrian, we do not take them into account for the reason that the radar signal of pedestrian is poor, which is consistent with [11].

The main contributions of this paper are summarized as follows:

- A spatial attention fusion (SAF) block to integrate radar data and vision data is proposed, which is built on the FCOS vision detection framework;
- We generate the 2D annotations of nuScenes dataset for model training and inference;
- An improved generation model to transform the radar information to an RGB image is proposed, which is used as the input data of our proposed SAF block;
- A lot of experiments to select the optimal hyperparameters involved in the radar image generation model are carried out;

The rest of the paper is organized as follows. Section 2 reviews the related work. In Section 3, details of improved radar image generation model and the labeling process of 2D annotations in vision images are clarified. Then, implementation details about the SAF block for obstacle detection using mmWave Radar and Vision are given. Next, the training details and the numerical results are provided in Section 5. Finally, Section 6 concludes the paper.

## 2. Related Works

### 2.1. Object Detection by Vision Sensors

For the object detection by vision sensors in CNN, there are two kinds of detectors: two-stage detector and one-stage detector. The R-CNN [3] was first presented by integrating segmentation algorithms [40,41] into AlexNet [42] to sample region proposals. To improve the detection speed, SPPNet [5] proposed a spatial pyramid pooling layer to reuse the feature maps for region proposals. Similar to the SPPNet, the Fast R-CNN [4] reused the feature maps by integrating segmentation algorithms into feature level. In addition, a new ROI pooling layer was proposed to convert any valid region of interest into a feature map with a fixed spatial extent. Instead of using segmentation algorithms to generate object proposals, Ren et al. [6] proposed a region proposal network (RPN) upon the Fast R-CNN to produce object candidates, which was known as Faster R-CNN.

To achieve a fast running speed, the one-stage detectors [1,2,8,9,10] are proposed. The one-stage detectors conducted bounding box regression and classification over densely predefined regular object locations. In the work of [1], the YOLO was proposed to make bounding boxes and confidence scores prediction on grid cells, which were the sub-regions of camera coordinate. To improve the accuracy of one-stage detector, Liu et al. [2] discretized the output space of bounding boxes into a set of default boxes over different aspect ratios and scales per feature map location, which was called SSD. The SSD model can leverage multiple feature maps for object detection. However, the extreme foreground-background class imbalance during training was the main bottleneck to prevent one-stage detector from achieving state-of-the-art accuracy. In the paper of [8], Lin at al. introduced a new dynamically scaled cross entropy loss, where the scaling factor decayed to zero as confidence in the right correct class increased. In contrast to SSD, Lin et al. [7] developed a top-down architecture with lateral connection for building high-level semantic feature maps at all scales. The above mentioned one-stage detectors are all anchor-based detection model. Without anchors and region proposals, Tian et al. [10] proposed a fully convolutional one-stage object detector to solve object detection in a per-pixel prediction, analogue to semantic segmentation. More importantly, the FCOS [10] is not only superior than two-stage algorithm Faster R-CNN in accuracy and speed, but also easy to understand.

Although the detection methods [6,7,10] using vision sensor based on CNN have achieve state-of-the-art performance in VOC [43] and MS COCO [38] dataset, these object detection models have limited performance when obstacles have blurred appearance because of bad weather or the size of obstacle is small. In addition, the fusion scheme using radar and vision sensor is a promising direction to improve the detection performance in autonomous system. Thus, in this paper, we use the FCOS detection framework as detection backbone for the fusion of radar and vision sensor.

### 2.2. Object Detection by Decision Level Fusion

For the decision level fusion using mmWave radar and vision sensor, Langer et al. [12] described an integrated mmWave radar and vision sensor system for autonomous on-road navigation. To accurately detect and classify obstacles with respect to the danger, they also proposed a vision-based lane-keep system. To combine different sensors (laser, radar and vision), Coué et al. [13] demonstrated the interest of using probabilistic reasoning techniques to address the challenging multi-sensor data fusion problem. However, the fusion method proposed in [13] depends on the specific combination of sensors devices, algorithms or application systems. It should notice that there will be many new sensors and upgraded recognition algorithms, which will cause a huge of combinations. To solve the problem, Kawasaki et al. [14] introduced a Bayesian Network, which can make fusion dynamically.

In contrast to [13,14], Ćesić et al. [15] proposed a decision fusion scheme on the special Euclidean group using radar and stereo vision sensor [16]. For multi-object tracking, the joint integrated probabilistic data association (JIPDA) filter [44] was used, which run on matrix Lie groups. To take the advantages of both stereo cameras and radar, Wu et al. [17] fused detection results from different sensors by extended Kalman filtering (EKF), which can addresses the problem of accurately estimating the location, size, pose, and motion information of a threat vehicle. Due to the poor radar signal in pedestrians, Chavez-Garcia et al. [18] only fused the LiDAR and vison sensor for final decision. However, three kinds of sensors including radar, LiDAR and vision were used for vehicle detection in [18]. To recover the 3D velocity associated with each target in the field, Zhong et al. [19] built a 3D velocity synthesis model to estimate velocity vectors using the fusion results of radar and vision sensor. In contrast to Kalman filter and Bayesian fusion methods, Kim et al. [20] proposed an information fusion method. Moreover, their radar signal processing algorithm inherited from [45].

Because radar and vision sensors are asynchronous, it is very crucial to make temporal data alignment before any fusion operations. In [21], Steux et al. implemented a set of filters to interpolate radar and vision outputs for data alignment. In contrast to [21], Streubel et al. [22] introduced a fusion time slot, where all targets of camera and radar appearing in the same time slot were validated in a measurement-to-measurement data association before fusion and tracking. For the dataset nuScenes [37] used in this paper, it designs a sensor synchronization system to collect data, which generally yields good data alignment results.

To help visually impaired people, Long et al. [23] proposed a fusion of mmWave radar and RGB-depth sensors for assisted navigation. They used MeanShift [46] algorithm to detect objects in the depth images. In addition, the distance of the detection object was decided by the average depth in the interest region. In the enhanced version [24], they introduced some improvements to vision data processing, where the Mask R-CNN [47] was used for object detection.

### 2.3. Object Detection by Data Level Fusion

Among all the fusion schemes, the data level fusion scheme is the most cost-effective computing method. In general, the data level fusion scheme for object detection can be summarized as two-steps. In the first step, a target-list is generated from the radar sensor. The items in the list are hypotheses for the presents of obstacles. In the second step, hypotheses are proved by the vision system. In addition, all of methods with data level fusion are mainly different in implementation details of the second step. Milch et al. [25] used a flexible 2D prior model of silhouette shape to recognize and track pedestrians in vision image sequences. In the paper of [26], Bombini et al. proposed a vehicle detection algorithm based on symmetry, where the radar data provided the areas of interest. For autonomous vehicle safety consideration, the paper of [27] also added the guard rail into detection consideration.

For feature extraction and candidate classification involved in vision sensors processing, Kadow et al. [28] used the Harr-like filters as feature-extraction model. After extracting feature vectors by Haar-like model, an AdaBoost algorithm was applied for hypothesis generation. In addition, the hypothesis was verified by an evolutionarily optimized and biologically motivated vehicle recognition system. To enhance the representation ability of Haar-like model in autonomous driving, Haselhoff et al. [29] made a set of tests about histogram equalization, gray-level variance and contrast normalization. With respect to the test images, the contrast normalization has the best performance. In general, the pixels from each candidate region may contain some information unrelated to the object, such as properties of a surface behind the object or noise. To deal with that, Ji et al. [30] implemented sparse coding by using orientation-selective filters for feature extraction, which were generated from images of nature henceforth called natural images through the Lobe Component Analysis (LCA) algorithm [48]. In addition, a multi-layer in-place learning network was used to distinguish sparse representations of different objects.

Because the ROI defined by radar signal in the image plane is much bigger than the projected object at the measured position. In addition, the object height cannot be measured by the radar. Serfling et al. [31] fused raw sensor data using a cascaded classifier, which was a robust and reliable pedestrian recognition system at night. In contrast to [31], Kato et al. [32] introduced a motion stereo technique with the help of the distance measured by the radar to find the boundaries of obstacles.

To make the radar-vision coordinate calibration, Wang et al. [33] proposed an experimental method for radar-vision point alignment using easy operation with no reflection intensity of radar, and special tool requirements was put forward. To simplify the calibration between radar and vision sensor, Guo et al. [34] taken an easy way to get the transform matrix. In this paper, our model training dataset—nuScenes [37] has provided the transform matrixes among all sensor units. Thus, we do not need to make sensor calibration step.

### 2.4. Object Detection by Feature-Level Fusion

Compared to the vision sensor-based detection methods, detection model using the feature fusion scheme can significantly improve the detection success rate of smaller vehicles in the bad weather. In the work of [11], Simon et al. was the first one to use CNNs network for the fusion of mmWave radar and vision information, where the detection backbone was based on the SSD [2] framework. The feature-extraction backbone of vision data was from ResNet blocks [49], which were an 18-layer ResNet variant. To make the radar point useable in the SSD framework, they proposed a radar image generation model to convert the radar points to images, which were easily used as the input data of convolutional neural network. In addition, their prediction head was built on the feature maps after the fusion between radar and vision sensor. Thus, there were two input branches and one output branch. For the radar images, they made experiments using the radar data in two ways. The first one was that an additional branch for the generated radar images was added in the SSD detection framework. Then, the feature map of radar image were concatenated with the second output of the ResNet branch. As for the second method, the same additional branch were used but without the max-pooling layer. After that, an element-wise addition was used to fuse the features after the first ResNet branch. Based on the evaluation results, they found that the small, medium and large vehicles can benefit from the feature fusion scheme. Moreover, the second fusion method was better than the first fusion method in all metrics. Compared to the decision level scheme and data level fusion scheme, the consuming of computing resources increased by the feature-level fusion scheme can be ignored. However, the feature fusion blocks used in [11] were very simple, and the sparsity of radar point was not considered to be well.

In contrast to [11], John et al. [35] proposed a novel deep learning-based sensor fusion framework, known as the “RVNet”. The RVNet was a single shot object detection network with two input branches and two output branches. For speed consideration, they built upon the YOLO [1] framework. The two input branches of RVNet were associated with the monocular camera and radar sensor. For the output two branches, the proposed network contained separate branches for small obstacles and big obstacles, respectively. For the fusion between radar feature maps and vision feature maps, the RVNet used the concatenation neural network layer, which was the same as the first method used in [11]. However, the two output branches in the RVNet can introduced more weights for model learning, which was more susceptible to overfitting. In addition, the consuming of computing resources in the RVNet was larger than the fusion detection model proposed by [11].

To decide which level the fusion of radar data and vision data was the most beneficial for detection performance, the work of [36] proposed a CameraRadarFusionNet (CRF-Net) to automatically learn that knowledge. Also, inspired by the Dropout layer in the deep learning method, they introduced a new training strategy to focus the learning on a specific sensor type, which was called BlackIn. In practice, the work of [36] used the nuScenes dataset for model training, validation and inference, which was the same as ours. In addition, to filter noise involved in radar points, they introduced a ground-truth noise filter to augment the performance of fusion detection model. By the way, their neural network architecture built on RetinaNet [8] as implemented in [50] with a VGG backbone [51]. From their evaluation results in nuScenes test dataset, the baseline image network was 43.47% on average precision, while the CRF-Net was 43.95% on average precision. In conclusion, the improvement of detection performance was limited. For feature fusion block used in [36], the element-wise addition was adopted as the fusion operation in the CRF-Net, which was different from [35].

In brief, the feature fusion blocks used in the published sensor fusion detection methods are very simple. In this paper, we propose a spatial attention fusion (SAF) block to learn the relationship between radar data and vision data, which can improve the detection performance in small, medium and large scales.

### 2.5. Object Detection by Mixed Fusion

In addition, more than one fusion scheme is used in some algorithms [52,53,54,55]. Lindl et al. [52] proposed a three-level early fusion scheme to fuse a far infrared imaging device, a laser scanner and several radar sensors. They adopted a data association algorithm from Hopcroft to fuse the detection results from different sensors. In the work of [53], the ROIs used in vision sensors were generated from laser points. In addition, the detection results were merged from radar, camera and LiDAR. The decision level fusion and data level fusion were used at the same time. Wang et al. [54] proposed a vehicle detection and tracking algorithm using decision level fusion and data level fusion as well. First, the radar signal provided the localization and size of ROI for vision sensor. Then, the object trajectories generated from vision and radar were compared and verified to confirm whether the detection and tracking were valid. In the work of [55], a different feature-extraction model and classification algorithm were used to process the data of vision sensor.

## 3. Training Dataset

In this paper, we use nuScenes dataset [37] for model training. This dataset is the first one to carry the full autonomous vehicle sensor suite: 6 cameras, 5 radars and 1 LiDAR, all with full 360-degree field of view. For the details of different sensors, please refer to the paper of [37]. In general, the camera has a 1600×900 resolution, and the radar is a 77GHz FMCW mmWave radar. In addition, the radar’s detection range is ≤250m. The sensor setup of nuScenes data collection platform can be found in “Figure 3” of their paper [37]. As for the sensor calibration, with the help of tools like laser liner and calibration target boards, they express extrinsic coordinate of each sensor to be relative to the *ego frame*, which is the midpoint of the rear vehicle axle. For our model training, only the front camera and front radar are used to build the feature fusion detection model, which is based on the FCOS [10] framework. Basically, a single radar point has 18 physical states defined in nuScenes [37] dataset, including position, velocities, rcs, etc. We only use the position and velocity information, while the other physical states are not used.

**Figure 4:**
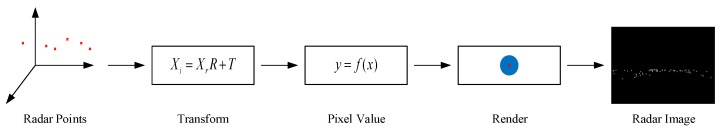
The radar image generation model.

The original radar points cannot be directly used by CNN network. Inspired by the paper of [11], we redesign the radar image generation model and make some changes about how to convert the physical states to pixel values in radar image plane. The whole generation process is displayed in Figure 4. First, the radar points in 3D radar coordinate are transformed into camera coordinate of front camera by the function $X_{i}=X_{r}R+T$. The input data is $X_{r}$, which represents the 3D position information in radar coordinate. Using the calibration matrixes, R (rotation matrix) and T (translation matrix), we can get the radar point location $X_{i}$ in the camera coordinate. After the transformation, we convert the depth *d*, longitudinal velocity $v_{x}$ and lateral velocity $v_{y}$ to a real pixel value in different channels (R,G,B). The three convert equations are defined as follows:

(1) $$R=\frac{128d}{250}+127\;,\;G=\frac{128\left(v_{x}+20\right)}{40}+127\;,\;B=\frac{128\left(v_{y}+20\right)}{40}+127\;.$$

For the unknown areas with no radar points, their pixel values in different channels are all 0. In the next generation step, a solid circle with a radius of *r* pixels and color provided in the Equation (1) is rendered in the radar image, where the center of the solid circle is the location of radar point transformed into the camera coordinate. As shown in Figure 4, the blue solid circle is the rendering result of single radar point. After processing all radar points in the location range of front camera image plane, the generated radar image is born. The size of it is the same as the front camera, which also has the resolution 1600×900.

**Figure 5:**
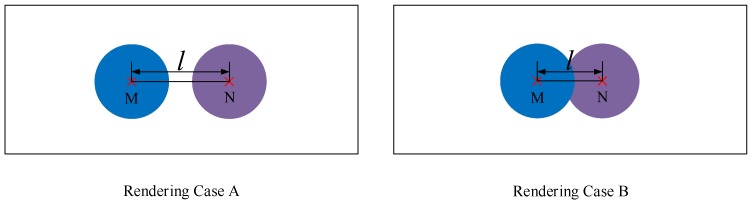
The two kinds of rendering cases involved in rendering process of radar image generation model.

By the way, there are two kinds of rendering cases involved in radar image generation model, which are shown in Figure 5. If the distance *l* between radar point M and radar point N (which have been transferred into image plane) is more than two times of rendering radius *r*, the rendering process is depicted as Rendering Case A. When the distance *l* between radar point M and radar point N is less than two times of rendering radius *r*, there are overlapping areas between them if they are rendered by the rendering radius *r*. In that case, we apply a different rendering rule: If the depth $d_{M}$ of radar point M is less than the depth $d_{N}$ of radar point N, the rendering process is depicted in Rendering Case B. Basically, the rendering rule of Rendering Case B is inspired by the “near the far smaller” law. Thus, when there are overlapping areas between two radar points, the radar point with smaller depth should occupy more areas. In addition, the generated radar images must be saved as png format. If they are saved as jpg format, some of noises may be introduced, which has been confirmed by the experimental result.

For the 2D annotations of obstacles, we do not use the tool provided by nuScenes dataset [37] to convert the 3D bounding boxes to 2D annotations. Because the 2D annotations converted by 3D annotations are not well labeled, which can be noticed in the Figure 3. Instead, we generate the 2D annotations by enhanced version of FCOS with the ResNet-101. In addition, all generated 2D annotations are roughly examined and adjusted by us.

## 4. Proposed Algorithm

**Figure 6:**
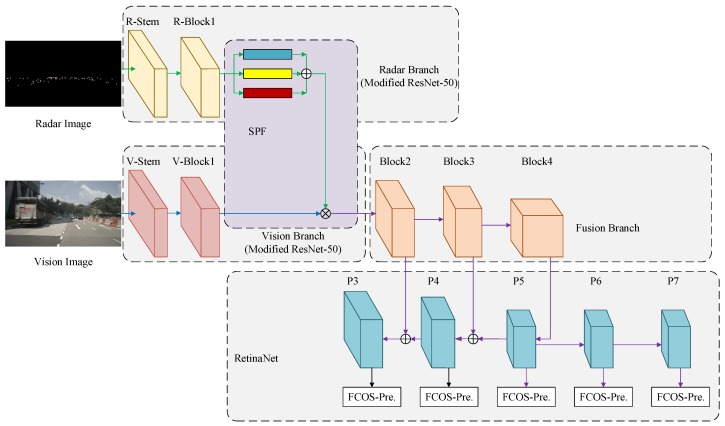
The proposed spatial attention fusion-based fully convolutional one-stage network (SAF-FCOS) for obstacle detection. In addition, the FCOS-Pre. block stands for the prediction head used in FCOS detection framework. The P3 stands for Phase 3.

In this section, we give a comprehensive introduction about our proposed spatial attention fusion-based fully convolutional one-stage network (SAF-FCOS) for object detection. The proposed SAF-FCOS uses a SAF block to merge the feature maps of radar and vision sensor. To analyze the proposed SAF block, we make lots of comparison experiments to confirm its superiority. The overall detection framework is depicted in Figure 6.

### 4.1. Detection Framework

Our proposed feature fusion detection model is based on FCOS framework [10]. It mainly consists five parts: Radar Branch (feature-extraction model of radar image), Vision Branch (feature-extraction model of vision image), SAF block, Fusion Branch (feature extraction based on fused feature maps), and RetinaNet [8] (the FCOS prediction head is used).

The radar branch is a modified ResNet-50 [49]. It has two convolution blocks: R-Stem and R-Block1. The R-Stem is the original stem module of ResNet-50 to process the input data. The R-Block1 is similar to the first stage in ResNet-50, but it has only a residual block compared to 3 residual blocks in ResNet-50. The reason is that if three residual blocks is introduced in the first stage as ResNet-50, the whole detection model cannot be updated by stochastic gradient descent easily. In addition, the output values of loss function are always “nan” in early iterations. Worse still, we cannot train the SAF-FCOS model easily even if we restart the training code multiple times. However, when we set the number of residual blocks in the first stage as 1, we may need to make a few tries to restart the training code so as to help the detection model to get rid of “nan” in losses. We think that it is because more residual blocks are not suitable for sparse radar images. Moreover, less residual blocks can save computing resources. As for the feature extraction of vision image, it also has two operation blocks: V-Stem and V-Block1, which are the same as the stem module and the first stage block in ResNet-50.

For the SAF block, it encodes the radar image’s feature maps to a spatial attention weight matrix. Then, the feature maps extracted by vision sensor are re-weighted by the spatial attention matrix along all channels. After that, the fused feature maps from radar and vision sensors are extracted by Block2, Block3 and Block4 in Fusion Branch to get multi-scale feature maps, where all of the blocks are the same stages in ResNet-50 backbone, which is used in the FCOS [10] framework (I recommend to read the source code of FCOS to understand the details of the whole framework. In addition, the python file resnet.py in the folder of “fcos_core/modeling/backbone/” should be the first for start.).

To achieve a superior detection performance, the FCOS [10] version of RetiaNet [8] is used for final result prediction, which is shown in the final part of Figure 6. In addition, the loss function is defined as FCOS [10]:

(2) $$L\left(c_{i},t_{i}\right)=\frac{1}{N_{pos}}\sum_{i}L_{cls}\left(c_{i},c_{i}^{*}\right)+\frac{\lambda }{N_{pos}}\sum_{i}{𝟙}_{c_{i}^{*}>0}L_{reg}\left(t_{i},t_{i}^{*}\right)\;.$$

where $c_{i}$ is the predicted class label and $c_{i}^{*}$ is the ground-truth label for a specific location *i* in a feature map. In addition, $t_{i}$ is the predicted bounding box, which is assigned a ground-truth bounding box $t_{i}^{*}$. In addition, the $L_{cls}$ is focal loss as in [8] and $L_{reg}$ is the IOU loss as in UnitBox [56]. $N_{pos}$ denotes the number of positive samples and λ being 1 in this paper is the balance weight for $L_{reg}$. ${𝟙}_{c_{i}^{*}>0}$ is the indicator function, being 1 if $c_{i}^{*}>0$ and 0 otherwise.

### 4.2. Spatial Attention Fusion

As we all know, the radar points reflect physical states of surroundings in autonomous vehicle. Thus, it is effective to use radar points as gate cells to control the information flow extracted from vision sensor. We hope that the information flow of small objects and blurred objects can be enhanced, to increase the recall rate in detection. As for the easy classified objects, the radar point can also have a positive influence. More importantly, the areas without any radar point are also considered by the proposed fusion detection method, which is different from the data level fusion scheme.

Based the analysis mentioned above, we propose a spatial attention fusion (SAF) block to generate a 2D matrix to re-weight the vision branch’s feature maps along all channels.

**Figure 7:**
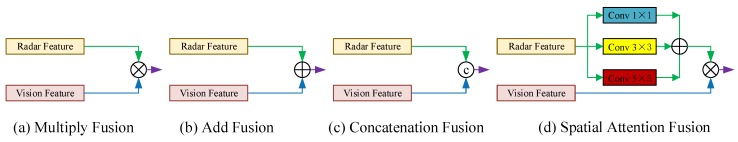
Different fusion blocks in feature fusion scheme. From the left to right: Multiply Fusion (MUL) Block, Element-Wise Add Fusion (ADD) Block, Concatenation Fusion (CAT) Block and Spatial Attention Fusion (SAF) Block.

The overall structure of proposed SAF is illustrated in Figure 7d.

**Figure 8:**
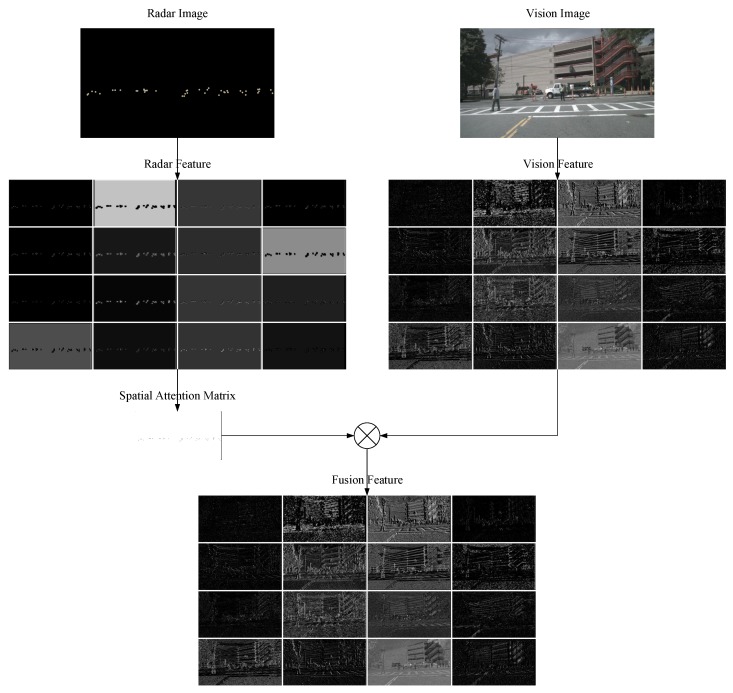
The visualization results about part of radar feature, part of vision feature, spatial attention matrix and part of fusion feature in SAF-FCOS. The channels of feature maps about the radar feature, vision feature and fusion feature are all 256. Due to space constraints, we only select 16 of them for visualization.

Our proposed SAF consists of three groups of convolution layers to extract spatial attention matrix. The configurations in layer “Conv 1×1” mean kernel size 1×1×256×1, stride (1, 1), padding [0, 0]. As for the layers of “Conv 3×3” and “Conv 5×5”, the configurations are {3×3×256×1, (1, 1), [1, 1]} and {5×5×256×1, (1, 1), [2, 2]}, respectively. Considering the configurations in the three convolution layers, the number of channel in the radar feature map is reduced to 1, while the output attention matrix has the same height and width as vision feature map. To introduce three different kinds of convolution layers, we hope the generated attention matrix has multi-scale receptive fields to learn the radar points’ representation and the relationship of surroundings, which is used as reasonable attention map to control or enhance the information flow within the vision sensor. As shown in Figure 8, the visualization results about part of radar feature, part of vision feature, spatial attention matrix and part of fusion feature in SAF-FCOS are displayed.

For other fusion blocks shown in Figure 7, the add fusion and concatenation fusion are first evaluated in [11]. After that, the work of RVNet [35] only makes experiments using concatenation fusion, while the add fusion is used in the work of [36]. We think that the fusion blocks used in the three mentioned methods are not the most suitable for feature fusion, because the radar features and vision features are not homogeneous, and the characteristics of radar signal are ignored. We also think that our proposed SAF is similar to the data level fusion implemented in feature maps, which is learnable with training dataset. To make fully comparisons among different fusion blocks, we also introduce a new simple Multiply Fusion block and make a lot of experiments as displayed in Section 5.

## 5. Experimental Validations

In this section, the proposed SAF-FCOS is evaluated on the autonomous driving dataset nuScenes [37], which is the first dataset to provide radar data. For object detection, there are more than 6k radar-vision image pairs for evaluation. We train SAF-FCOS using the union of 700 scenes of training data and 150 scenes of validation data, which has a total of 34149 radar-vision image pairs. We use the standard MS COCO [38] evaluation metrics in all experiments, including $AP^{IoU=0.50}$, $AP^{IoU=0.75}$, AP (averaged over Intersection-over-Union (IoU) values from 0.50 to 0.95), and $AP^{small}$, $AP^{medium}$, $AP^{large}$ to assess the performance of SAF-FCOS with different IoU thresholds and scales. Moreover, the max detections are set 100 in all average precision metrics. Here the metrics are also shown as AP(100), $AP^{0.50}$(100), $AP^{0.75}$(100), $AP^{s}$(100), $AP^{m}$(100), $AP^{l}$(100) for simplicity. Similarly, the performance metrics of average recall in different max detections and scales are shown as AR(1), AR(10), AR(100), $AR^{s}$(100), $AR^{m}$(100), $AR^{l}$(100), where the IoU threshold values are from 0.50 to 0.95.

At first, the implementation details about model training and inference are shown in Section 5.1. Then we compare the detection results of SAF-FCOS (using radar data and vision data) with FCOS (only using vision data) in Section 5.2. To evaluate the different fusion blocks (mentioned in Figure 7), we replace the SAF block in SAF-FCOS model and do lots of comparison experiments using the same training configurations, which are shown in Section 5.3. In Section 5.4, we give discussions about what is the most suitable number of rendering radius of radar points in radar image generation model for SAF-FCOS training and inference. Next, we introduce different SAF configurations to make clear about the role of convolution layers in the proposed SAF block in Section 5.5. Finally, the proposed SAF block is adopted to Faster R-CNN [6] detection framework to exhibit its generalization.

### 5.1. Implementation Details

We implement SAF-FCOS on the PyTorch [57] platform and train the model on 8 NVIDIA GeForce GTX 1080Ti GPUs with 12GB memory. The Vision Branch and Fusion Branch used in SAF-FCOS is first pre-trained on ImageNet [58] dataset and all the blocks are finetuned on the nuScenes [37] dataset. Moreover, the weights of R-Stem, R-Block1, layers in RentinaNet are random initialization as the FCOS. In addition, the layers of V-Stem and V-Block1 are not updated in model training. By default, we train SAF-FCOS using the Stochastic Gradient Descent (SGD) with a momentum of 0.9 and a weight decay of 0.0001 for 40k iterations in total. The learning rate is initialized to 0.01. The constant warmup scheme [49] for learning rate is adopted in the first few iterations of training. The batch in each iteration is 16 radar-vision image pairs. Moreover, the weights of SAF is initialized by MSRA initialization method [59]. In model training, the input radar images and vision images are resized to ensure that their shorter side being 800 and their longer side less or equal to 1333.

### 5.2. Detection Comparisons

The FCOS [10] detector is anchor box free, as well as proposal free. It has superior performance in MS COCO dataset [38]. In this paper, we propose a SAF block using the FCOS framework for obstacle detection. The SAF consists of three groups of convolution layers to extract spatial attention matrix. The attention matrix can be used to control or enhance the information flow within the vision sensor.

**Figure 9:**
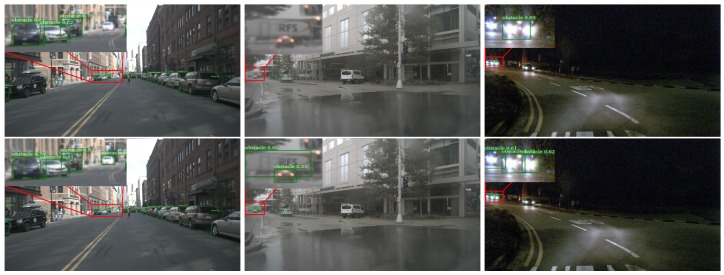
The detection performance comparison between FCOS [10] and SAF-FCOS in sunny, rainy and night. The images in the top row are detection results by FCOS, and the images in the bottom row are detection results by SAF-FCOS. The comparisons demonstrate that the proposed SAF-FCOS has a better performance in small and far away obstacles.

We first compare the SAF-FCOS with the FCOS detection framework, which is training only by vision images. The qualitative evaluation results are depicted in Figure 9. From the left to the right, there are three different scenes, sunny day, rainy day and night. The detection results in the top row are coming from FCOS, while the bottom is provided by our proposed SAF-FCOS. Compared to the FCOS, the SAF-FCOS can detect small and far away obstacles, which are very curial for autonomous vehicle to plan the path. We think that the improvement of detection is attributed to more sensor information involved in the detection process. Thus, we think that the sensor fusion is a promising direction to break through the bottleneck of detection algorithms, which only rely on the vision sensor.

For quantitative analysis, we report the average precision and average recall results in different metrics as MS COCO [38] using the nuScenes [37] test dataset, which are shown in Table 1. The feature-extraction backbone used in FCOS and SAF-FCOS is part of ResNet-50 [49]. The test scale of input radar images and vision images are 800, which means the shorter side of image is resized to 800 pixels. From Table 1, our proposed SAF-FCOS has outperformed the FCOS in all metrics. Specifically, in the AP(100), $AP^{0.50}$(100) and $AP^{0.75}$(100), the SAF-FCOS has made gains about 7.7%, 3.8% and 8.6%, respectively. Based on that, we can conclude that the SAF-FCOS generates more tighter bounding boxes than FCOS. Moreover, the SAF-FCOS outperforms the FCOS by 9.3% in $AP^{s}$(100), which is larger than the gains of $AP^{m}$(100) 7.4% and $AP^{l}$(100) 6.4%. It confirms that the detection performance of small obstacle benefits from the radar sensor. More importantly, the SAF-FCOS is also superior than FCOS in all average recall metrics. With the help of SAF bock in the feature fusion scheme using radar and vision, the low recall rate in the data level fusion scheme is avoided. Because the recall rate in the data level fusion scheme depends on the number of effective radar points, which are associated with obstacles [28].

**Figure 10:**
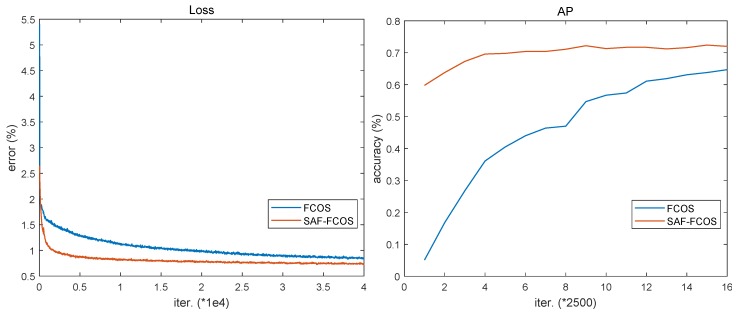
The loss and AP plots about FCOS and SAF-FCOS in different iterations.

In addition, there is an interesting experimental phenomenon in model training. As shown in Figure 10, the training loss of SAF-FCOS decreases more rapidly than FCOS during whole iterations. Furthermore, the AP accuracy plot of SAF-FCOS in test dataset is soon stabilized compared to FCOS, and the AP accuracy of SAF-FCOS is always larger than FCOS during 40000 iterations.

### 5.3. The Comparison of Different Fusion Blocks

To evaluate different fusion blocks mentioned in Section 4.2, we train different fusion models based on the FCOS detection framework and make predictions on the nuScenes dataset. The training configurations and implementation details are the same as SAF-FCOS. For the concatenation fusion block, the channels of feature map coming from radar branch and vision branch are both 256. After that, we add a convolution layer with the configuration 1×1×512×256 in kernel size to reduce the channels of the output of the concatenation fusion block. Finally, the channels of input feature maps of Fusion Branch are all 256 in different feature fusion schemes, which are consistent with the SAF-FCOS.

As shown in Table 2, our proposed SAF-FCOS is superior to other fusion detection models in all average precision metrics and average recall metrics. Basically, the CAT-FCOS has the worst performance, which is lower about 12.6% in AP compared to SAF-FCOS. Moreover, the performance of CAT-FCOS and MUL-FCOS is close. Similar to [11], the element-wise add fusion scheme outperforms the concatenation fusion scheme. Of course, the SAF-FCOS has made a gain about 8.2% in AP than ADD-FCOS. For the fusion blocks add, concatenation and multiply, we think that they are not well-designed fusion blocks for radar and vision sensor. However, our proposed SAF fusion block does not need to face the heterogeneous features, and it generates an attention matrix to control or enhance the information flow within the vision sensor, which is inspired by the properties of radar signal.

### 5.4. The Comparison of Different Rendering Radius

How do the different rendering radiuses in radar images influence the detection performance of SAF-FCOS model? To reveal the mystery, we generate six groups of radar images using different rendering radiuses, which are r=1, r=3, r=5, r=7, r=9 and r=11. The training configurations in all experiments are the same except the setting of rendering radius in radar image generation model. The SAF block is made up of layers “Conv 1×1”, “Conv 3×3” and “Conv 5×5”. Moreover, we conduct six comparison experiments on the nuScenes validation dataset [37], which are used to select the optimal rendering radius.

The obstacle detection comparison results in all average precision metrics and average recall metrics are shown in Table 3. Considering both average precision metrics and average recall metrics, the SAF-FCOS using radar images with rendering radius r=7 has the best detection performance. Moreover, we should notice that the detection performance of r=1 is very close to r=7. As a result, the radar images with r=7 using for SAF fusion method are the most suitable. In the following experiments, we use the radar images with the rendering radius r=7 in SAF-FCOS.

### 5.5. The Comparison of Different SAF Configurations

To make clear about the influence of different configurations to generate SAF blocks, we make lots of experiments. The fundamental idea to build the SAF block is to explore the permutations about different kinds of convolution layers. In this paper, all of permutations can be classified into five categories: without SAF, SAF made by a specific convolution layer, SAF made by two kinds of convolution layers, SAF made by three kinds of convolution layers and SAF made by four kinds of convolution layers. For strict comparisons, the training configurations and implementation details in different SAF configurations using FCOS detection framework are the same except the SAF. The feature-extraction backbone is also ResNet-50 [49], and test scale is 800.

All detection performance with different SAF configurations are displayed in Table 4. Base on the comparison results, the FCOS using the SAF made by a specific convolution layer with 3×3, 5×5, or 7×7 outperforms the FCOS in all average precision metrics, which has no SAF fusion block. Moreover, the SAF built by 1×1 convolution layer is not as well as FCOS in all metrics. We think that the receptive field of 1×1 convolution layer is too small to generate a meaningful attention matrix, which is not suitable to control or enhance the information flow in vision sensor.

As for the SAF made by two kinds of convolution layers, the detection performance of SAF with 1×1 and 3×3 convolution layers is superior than SAF with 1×1 or SAF with 5×5. However, the other two versions of SAF using two kinds of convolution layers are not support that. For example, SAF with 3×3 and 5×5 is not as good as SAF with 3×3 or SAF with 5×5. When the SAF is made up of three different convolution layers, the detection performance is superior than one convolution layer used and two convolution layer used (except the case of SAF made by the 5×5 convolution layer). We think that three groups of convolution layers can extract multi-scale receptive fields, which are essential to generate a useful spatial attention matrix to fuse the information of radar and vision sensor.

Moreover, when the SAF is made up of 1×1, 3×3, 5×5, and 7×7 convolution layers, the detection performance in AP (70.1%) is very close to the best SAF configuration AP (70.2%). However, we should notice that the SAF with the best AP still performs not as well as the SAF with four different convolution layers in some metrics. Based on the strategy in MS COCO [38], the AP is used to select the best detection model for final decision. Therefore, the fusion detection models in other sections for experiments use the SAF consisting 1×1, 3×3, and 5×5 convolution layers.

### 5.6. The Evaluation of SAF-Faster R-CNN

To confirm the generalization of proposed SAF feature fusion method, we introduce a different detection framework Faster R-CNN [6]. Similar to FCOS, the feature-extraction backbone in Faster R-CNN is ResNet-50 [49]. As shown in Figure 6, we replace the prediction block with the one used in Faster R-CNN framework to build another feature fusion detection model SAF-Faster R-CNN. The rendering radius of radar images used in model training and inference is r=7. The SAF configurations are three groups of convolution layers: 1×1, 3×3, and 5×5. By the way, the training configurations of SAF-Faster R-CNN inherit from SAF-FCOS.

The comparison results between Faster R-CNN and SAF-Faster R-CNN are shown in Table 5. As usual, the proposed SAF feature fusion method can still enhance the detection performance of Faster R-CNN, which is a very favorable evidence to support the excellent generalization of proposed SAF fusion model. Of course, the improvement of SAF-Faster R-CNN compared to Faster R-CNN is not as large as FCOS. We attribute that the training configurations of SAF-Faster R-CNN inherit from SAF-FCOS, which is not the most suitable for SAF-Faster R-CNN detection model.

## 6. Conclusions

In this paper, a novel feature fusion method SAF using mmWave radar and vision sensor is proposed to detect obstacles. In contrast to the other fusion blocks in feature fusion scheme, the proposed SAF leverages the radar features to generate an attention matrix to control or enhance the information flow within the vision sensor. Compared to other fusion scheme in different levels, the fusion detection framework using SAF can train the detection model by an end-to-end manner. In model training, we transfer radar points to radar images, which are the same size of vision images. Compared to the vision detection framework FCOS [10], the proposed SAF-FCOS (using radar data and vision data) has a better detection performance in all scales than FCOS. More importantly, the improvement of $AP^{s}$(100) is larger than other scales.

In the future work, we decide to extend the SAF feature fusion method into multi-cameras and multi-radar sensors, which can provide 360° sensor coverage. To further improve the detection success rate, we think the multi-object tracking and obstacle detection model can support each other. Moreover, the decision fusion scheme can be integrated with the SAF to further improve the detection performance. 

## Figures and Tables

**Table 1:** Obstacle detection comparisons using different sensors on the nuScenes test dataset [37]. The top sub-table is about average precision metrics. In addition, the bottom sub-table is about average recall metrics. The bold in every column indicates the highest score.

Model	Backbone	Scale	AP(100)	${\mathrm{AP}}^{0.50}$(100)	${\mathrm{AP}}^{0.75}$(100)	${\mathrm{AP}}^{s}$(100)	${\mathrm{AP}}^{m}$(100)	${\mathrm{AP}}^{l}$(100)
FCOS	ResNet-50	800	64.7	86.2	70.9	46.0	62.7	76.7
SAF-FCOS	ResNet-50	800	**72.4**	**90.0**	**79.3**	**55.3**	**70.1**	**83.1**
**Model**	**Backbone**	**Scale**	AR **(1)**	AR **(10)**	AR **(100)**	${\mathrm{AR}}^{s}$ **(100)**	${\mathrm{AR}}^{m}$ **(100)**	${\mathrm{AR}}^{l}$ **(100)**
FCOS	ResNet-50	800	13.7	62.3	73.5	60.0	72.5	82.3
SAF-FCOS	ResNet-50	800	**14.5**	**68.4**	**79.0**	**69.6**	**77.4**	**86.5**

**Table 2:** Obstacle detection comparisons using different feature fusion blocks on the nuScenes test dataset [37]. The top sub-table is about average precision metrics. In addition, the bottom sub-table is about average recall metrics.

Model	Backbone	Scale	AP(100)	${\mathrm{AP}}^{0.50}$(100)	${\mathrm{AP}}^{0.75}$(100)	${\mathrm{AP}}^{s}$(100)	${\mathrm{AP}}^{m}$(100)	${\mathrm{AP}}^{l}$(100)
CAT-FCOS	ResNet-50	800	59.8	85.7	64.4	36.4	57.9	74.8
MUL-FCOS	ResNet-50	800	61.6	86.4	67.5	39.8	60.9	74.5
ADD-FCOS	ResNet-50	800	64.2	87.6	70.9	42.7	62.8	77.4
SAF-FCOS	ResNet-50	800	**72.4**	**90.0**	**79.3**	**55.3**	**70.1**	**83.1**
**Model**	**Backbone**	**Scale**	AR **(1)**	AR **(10)**	AR **(100)**	${\mathrm{AR}}^{s}$ **(100)**	${\mathrm{AR}}^{m}$ **(100)**	${\mathrm{AR}}^{l}$ **(100)**
CAT-FCOS	ResNet-50	800	13.3	58.4	68.1	48.7	66.6	80.6
MUL-FCOS	ResNet-50	800	13.4	60.1	70.2	52.6	69.3	80.9
ADD-FCOS	ResNet-50	800	13.7	61.9	71.9	55.1	70.9	82.5
SAF-FCOS	ResNet-50	800	**14.5**	**68.4**	**79.0**	**69.6**	**77.4**	**86.5**

**Table 3:** Obstacle detection comparisons using radar images with different rendering radiuses on the nuScenes validation dataset [37]. The top sub-table is about average precision metrics. In addition, the bottom sub-table is about average recall metrics.

Radius	Backbone	Scale	AP(100)	${\mathrm{AP}}^{0.50}$(100)	${\mathrm{AP}}^{0.75}$(100)	${\mathrm{AP}}^{s}$(100)	${\mathrm{AP}}^{m}$(100)	${\mathrm{AP}}^{l}$(100)
r=1	ResNet-50	800	70.1	**90.4**	**77.1**	52.3	68.4	81.0
r=3	ResNet-50	800	69.6	89.5	75.8	52.3	67.9	80.4
r=5	ResNet-50	800	64.5	87.1	70.0	47.1	63.2	75.2
r=7	ResNet-50	800	**70.2**	89.9	76.7	**54.3**	**68.6**	**80.6**
r=9	ResNet-50	800	69.7	89.9	75.7	52.8	68.5	79.8
r=11	ResNet-50	800	68.3	89.0	74.5	52.9	66.6	79.2
**Radius**	**Backbone**	**Scale**	AR **(1)**	AR **(10)**	AR **(100)**	${\mathrm{AR}}^{s}$ **(100)**	${\mathrm{AR}}^{m}$ **(100)**	${\mathrm{AR}}^{l}$ **(100)**
r=1	ResNet-50	800	12.8	64.5	76.9	66.6	75.6	84.9
r=3	ResNet-50	800	12.7	64.5	76.6	65.2	75.5	84.7
r=5	ResNet-50	800	12.4	60.2	73.3	59.9	73.2	80.8
r=7	ResNet-50	800	**12.7**	**65.2**	**77.4**	**66.8**	**76.3**	**85.0**
r=9	ResNet-50	800	12.6	64.1	76.7	65.5	76.0	84.2
r=11	ResNet-50	800	12.6	63.1	76.3	65.5	75.2	84.2

**Table 4:** The detection performance of average precision metrics and average recall metrics with different SAF configurations on the nuScenes validation dataset. The configuration of “1×1” stands for a convolution layer with kernel size 1×1×256×1, stride (1, 1), padding [0, 0]. As for the layers of “3×3”, “5×5” and “7×7”, the configurations are {3×3×256×1, (1, 1), [1, 1]}, {5×5×256×1, (1, 1), [2, 2]}, and {7×7×256×1, (1, 1), [3, 3]}, respectively.

SAF									
1×1	3×3	5×5	7×7	AP(100)	${\mathrm{AP}}^{0.50}$(100)	${\mathrm{AP}}^{0.75}$(100)	${\mathrm{AP}}^{s}$(100)	${\mathrm{AP}}^{m}$(100)	${\mathrm{AP}}^{l}$(100)
				60.7	84.6	65.6	43.6	58.7	72.7
✓				58.6	84.5	62.9	39.6	57.5	70.9
	✓			68.8	88.7	75.3	51.7	67.2	80.1
		✓		69.8	89.8	76.2	53.2	67.9	80.6
			✓	68.1	88.8	74.0	49.8	66.8	79.0
✓	✓			69.4	89.7	76.5	52.7	67.6	80.1
	✓	✓		67.0	88.3	72.8	49.0	65.2	78.5
		✓	✓	54.7	82.5	58.0	33.6	53.3	68.8
✓	✓	✓		**70.2**	**89.9**	**76.7**	**54.3**	68.6	**80.6**
	✓	✓	✓	69.8	89.6	75.9	53.6	67.7	**80.6**
✓	✓	✓	✓	70.1	**89.9**	**76.7**	52.4	**68.8**	80.3
**SAF**									
1×1	3×3	5×5	7×7	AR **(1)**	AR **(10)**	AR **(100)**	${\mathrm{AR}}^{s}$ **(100)**	${\mathrm{AR}}^{m}$ **(100)**	${\mathrm{AR}}^{l}$ **(100)**
				12.2	56.9	70.5	55.8	70.0	79.7
✓				12.0	55.5	68.4	50.6	68.1	78.5
	✓			12.6	63.7	77.0	64.9	76.2	85.0
		✓		12.6	64.9	77.2	66.0	76.4	84.8
			✓	12.6	63.0	76.2	64.5	75.5	83.8
✓	✓			12.7	63.8	76.6	66.5	75.1	84.7
	✓	✓		12.6	62.1	75.4	63.9	74.2	83.9
		✓	✓	11.7	52.8	64.7	45.7	63.8	76.8
✓	✓	✓		12.7	**65.2**	**77.4**	**66.8**	**76.3**	**85.0**
	✓	✓	✓	12.6	64.3	77.0	**66.8**	75.8	84.7
✓	✓	✓	✓	**12.8**	64.3	76.8	65.8	75.8	84.5

**Table 5:** Obstacle detection comparisons on the nuScenes test dataset [37] using the Faster R-CNN framework [6]. The top sub-table is about average precision metrics. In addition, the bottom sub-table is about average recall metrics.

Model	Backbone	Scale	AP(100)	${\mathrm{AP}}^{0.50}$(100)	${\mathrm{AP}}^{0.75}$(100)	${\mathrm{AP}}^{s}$(100)	${\mathrm{AP}}^{m}$(100)	${\mathrm{AP}}^{l}$(100)
Faster R-CNN	ResNet-50	800	71.9	89.3	78.5	55.1	68.9	83.5
SAF-Faster R-CNN	ResNet-50	800	**72.2**	**89.7**	**78.7**	**55.3**	**69.4**	**83.6**
**Model**	**Backbone**	**Scale**	AR **(1)**	AR **(10)**	AR **(100)**	${\mathrm{AR}}^{s}$ **(100)**	${\mathrm{AR}}^{m}$ **(100)**	${\mathrm{AR}}^{l}$ **(100)**
Faster R-CNN	ResNet-50	800	14.4	68.7	78.1	**68.9**	75.9	86.7
SAF-Faster R-CNN	ResNet-50	800	**14.5**	**68.7**	**78.5**	**68.9**	**76.6**	**86.8**

## References

[1] Redmon J., Divvala S., Girshick R., Farhadi A. You only look once: Unified, real-time object detection. Proceedings of the IEEE Conference on Computer Vision and Pattern Recognition (CVPR2016).

[2] Liu W., Anguelov D., Erhan D. SSD: Single shot multibox detector. Proceedings of the European Conference on Computer Vision Workshops (ECCV 2016).

[3] Girshick R., Donahue J., Darrell T., Malik J. Rich feature hierarchies for accurate object detection and semantic segmentation. Proceedings of the IEEE Conference on Computer Vision and Pattern Recognition (CVPR2014).

[4] Girshick R. Fast R-CNN. Proceedings of the IEEE International Conference on Computer Vision (ICCV2015).

[5] He K.M., Zhang X.Y., Ren S.Q., Sun J. (2015). Spatial pyramid pooling in deep convolutional networks for visual recognition. IEEE Trans. Pattern Anal. Mach. Intell..

[6] Ren S., He K., Girshick R., Sun J. (2017). Faster R-CNN: Towards Real-Time Object Detection with Region Proposal Networks. IEEE Trans. Pattern Anal. Mach. Intell..

[7] Lin T.L., Piotr D., Ross G., He K.M., Hariharan B., Belongie S. Feature pyramid networks for object detection. Proceedings of the IEEE Conference on Computer Vision and Pattern Recognition (CVPR2017).

[8] Lin T.Y., Goyal P., Girshick R., He K.M., Dollár P. Focal loss for dense object detection. Proceedings of the IEEE International Conference on Computer Vision (ICCV2017).

[9] Zhao X.T., Li W., Zhang Y.F., Chang S., Feng Z.Y., Zhang P. (2019). Aggregated Residual Dilation-Based Feature Pyramid Network for Object Detection. IEEE Access.

[10] Tian Z., Shen C.H., Chen H., He T. (2019). FCOS: Fully Convolutional One-Stage Object Detection. arXiv.

[11] Simon C., Will M., Paul N. Distant Vehicle Detection Using Radar and Vision. Proceedings of the 2019 International Conference on Robotics and Automation (ICRA2019).

[12] Langer D., Jochem T. Fusing radar and vision for detecting, classifying and avoiding roadway obstacles. Proceedings of the IEEE Conference on Intelligent Vehicles.

[13] Coué C., Fraichard T., Bessiere P., Mazer E. Multi-sensor data fusion using Bayesian programming: An automotive application. Proceedings of the IEEE 2002 Intelligent Vehicles Symposium.

[14] Kawasaki N., Kiencke U. Standard platform for sensor fusion on advanced driver assistance system using bayesian network. Proceedings of the IEEE 2004 Intelligent Vehicles Symposium.

[15] Ćesić J., Marković I., Cvišić I., Petrović I. (2016). Radar and stereo vision fusion for multitarget tracking on the special Euclidean group. Robot. Auton. Syst..

[16] Obrvan M., Ćesić J., Petrović I. Appearance based vehicle detection by radar-stereo vision integration. Proceedings of the Robot 2015: Second Iberian Robotics Conference.

[17] Wu S.G., Decker S., Chang P., Camus T., Eledath J. (2009). Collision sensing by stereo vision and radar sensor fusion. IEEE Trans. Intell. Transp. Syst..

[18] Chavez-Garcia R.O., Burlet J., Vu T.D., Aycard O. Frontal object perception using radar and mono-vision. Proceedings of the IEEE 2012 Intelligent Vehicles Symposium.

[19] Zhong Z.G., Liu S., Mathew M., Dubey A. (2018). Camera radar fusion for increased reliability in ADAS applications. Electron. Imaging.

[20] Kim D.Y., Jeon M. (2014). Data fusion of radar and image measurements for multi-object tracking via Kalman filtering. Inf. Sci..

[21] Steux B., Laurgeau C., Salesse L., Wautier D. Fade: A vehicle detection and tracking system featuring monocular color vision and radar data fusion. Proceedings of the IEEE 2002 Intelligent Vehicles Symposium.

[22] Streubel R., Yang B. Fusion of stereo camera and MIMO-FMCW radar for pedestrian tracking in indoor environments. Proceedings of the IEEE 19th International Conference on Information Fusion.

[23] Long N.B., Wang K.W., Cheng R.Q., Yang K.L., Bai J.S. Fusion of millimeter wave radar and RGB-depth sensors for assisted navigation of the visually impaired. Proceedings of the Millimetre Wave and Terahertz Sensors and Technology XI.

[24] Long N.B., Wang K.W., Cheng R.Q., Hu W.J., Yang K.L. (2019). Unifying obstacle detection, recognition, and fusion based on millimeter wave radar and RGB-depth sensors for the visually impaired. Revi. Sci. Instrum..

[25] Milch S., Behrens M. Pedestrian detection with radar and computer vision. Proceedings of the 2001 PAL Symposium—Progress in Automobile Lighting.

[26] Bombini L., Cerri P., Medici P., Alessandretti G. Radar-vision fusion for vehicle detection. Proceedings of the 3rd International Workshop on Intelligent Transportation.

[27] Alessandretti G., Broggi A., Cerri P. (2007). Vehicle and guard rail detection using radar and vision data fusion. IEEE Trans. Intell. Transp. Syst..

[28] Kadow U., Schneider G., Vukotich A. Radar-vision based vehicle recognition with evolutionary optimized and boosted features. Proceedings of the IEEE 2007 Intelligent Vehicles Symposium.

[29] Haselhoff A., Kummert A., Schneider G. Radar-vision fusion for vehicle detection by means of improved haar-like feature and adaboost approach. Proceedings of the IEEE 2007 15th European Signal Processing Conference.

[30] Ji Z.P., Prokhorov D. Radar-vision fusion for object classification. Proceedings of the IEEE 2008 11th International Conference on Information Fusion.

[31] Serfling M., Loehlein O., Schweiger R., Dietmayer K. Camera and imaging radar feature level sensor fusion for night vision pedestrian recognition. Proceedings of the IEEE 2009 Intelligent Vehicles Symposium.

[32] Kato T., Ninomiya Y., Masaki I. (2002). An obstacle detection method by fusion of radar and motion stereo. IEEE Trans. Intell. Transp. Syst..

[33] Wang T., Zheng N.N., Xin J.M., Ma Z. (2011). Integrating millimeter wave radar with a monocular vision sensor for on-road obstacle detection applications. Sensors.

[34] Guo X.P., Du J.S., Gao J., Wang W. Pedestrian Detection Based on Fusion of Millimeter Wave Radar and Vision. Proceedings of the 2018 International Conference on Artificial Intelligence and Pattern Recognition.

[35] John V., Mita S. RVNet: Deep Sensor Fusion of Monocular Camera and Radar for Image-Based Obstacle Detection in Challenging Environments. Proceedings of the 2019 Pacific-Rim Symposium on Image and Video Technology.

[36] Nobis F., Geisslinger M., Weber M., Betz J., Lienkamp M. A Deep Learning-based Radar and Camera Sensor Fusion Architecture for Object Detection. Proceedings of the 2019 Sensor Data Fusion: Trends, Solutions, Applications (SDF2019).

[37] Holger C., Bankiti V., Lang A.H., Vora S., Liong V.E., Xu Q., Krishnan A., Pan Y., Baldan G., Beijbom O. (2019). nuScenes: A multimodal dataset for autonomous driving. arXiv.

[38] Lin T.Y., Maire M., Belongie S., Hays J., Perona P., Ramanan D., Dollár P., Zitnick C.L. Microsoft coco: Common objects in context. Proceedings of the European Conference on Computer Vision (ECCV2014).

[39] FCOS Model. https://github.com/tianzhi0549/FCOS.

[40] Uijlings J.R.R., Van D.S., Koen E.A., Gevers T., Smeulders A.W.M. (2013). Selective search for object recognition. Int. J. Comput. Vis..

[41] Zitnick C.L., Dollár P. Edge boxes: Locating object proposals from edges. Proceedings of the European Conference on Computer Vision Workshops (ECCV 2014).

[42] Krizhevsky A., Sutskever I., Hinton G.E. Imagenet classification with deep convolutional neural networks. Proceedings of the Advances in Neural Information Processing Systems (NIPS 2012).

[43] Everingham M., Van Gool L., Williams C.K., Winn J., Zisserman A. (2010). The pascal visual object classes (voc) challenge. Int. J. Comput. Vis..

[44] Musicki D., Evans R. (2004). Joint integrated probabilistic data association: JIPDA. IEEE Trans. Aerosp. Electro. Syst..

[45] Sugimoto S., Tateda H., Takahashi H., Okutomi M. Obstacle detection using millimeter-wave radar and its visualization on image sequence. Proceedings of the 17th International Conference on Pattern Recognition (ICPR2004).

[46] Cheng Y.Z. (1995). Mean shift, mode seeking, and clustering. IEEE Trans. Pattern Anal. Mach. Intel..

[47] He K.M., Gkioxari G., Dollár P., Girshick R. Mask R-CNN. Proceedings of the IEEE International Conference on Computer Cision (ICCV2017).

[48] Weng J.Y., Zhang N. Optimal in-place learning and the lobe component analysis. Proceedings of the IEEE International Joint Conference on Neural Network.

[49] He K.M., Zhang X.Y., Ren S.Q., Sun J. Deep residual learning for image recognition. Proceedings of the IEEE Conference on Computer Vision and Pattern Recognition (CVPR2016).

[50] Fizyr Keras Retinanet. https://github:com/fizyr/keras-retinanet.

[51] Simonyan K., Zisserman A. (2014). Very deep convolutional networks for large-scale image recognition. arXiv.

[52] Lindl R., Walchshäusl L. (2006). Three-Level Early Fusion for Road User Detection. PReVENT Fus. Forum e-J..

[53] Chavez-Garcia R.O., Aycard O. (2015). Multiple sensor fusion and classification for moving object detection and tracking. IEEE Trans. Intell. Transp. Syst..

[54] Wang X., Xu L.H., Sun H.B., Xin J.M., Zheng N.N. (2016). On-road vehicle detection and tracking using MMW radar and monovision fusion. IEEE Trans. Intell. Transp. Syst..

[55] Liu X., Sun Z.P., He H.G. On-road vehicle detection fusing radar and vision. Proceedings of the IEEE 2011 International Conference on Vehicular Electronics and Safety.

[56] Yu J.H., Jiang Y.N., Wang Z.Y., Cao Z.M., Huang T. Unitbox: An advanced object detection network. Proceedings of the 24th ACM International Conference on Multimedia.

[57] Paszke A., Gross S., Massa F., Lerer A., Bradbury J., Chanan G., Killeen T., Lin Z., Gimelshein N., Antiga L. PyTorch: An imperative style, high-performance deep learning library. Proceedings of the Advances in Neural Information Processing Systems.

[58] Russakovsky O., Deng J., Su H., Krause J., Satheesh S., Ma S., Huang Z., Karpathy A., Khosla A., Bernstein M. (2015). Imagenet large scale visual recognition challenge. Int. J. Comput. Vis..

[59] He K.M., Zhang X.Y., Ren S.Q., Sun J. Delving deep into rectifiers: Surpassing human-level performance on imagenet classification. Proceedings of the IEEE International Conference on Computer Cision (ICCV2015).

